# Do specialist pulmonologists appropriately utilise thoracic surgery for drug-resistant pulmonary tuberculosis? A survey

**DOI:** 10.7196/SARJ.2018.v24i3.185

**Published:** 2018-09-07

**Authors:** G Alexander, R Perumal

**Affiliations:** 1 Department of Cardiothoracic Surgery, Inkosi Albert Luthuli Hospital and King Dinuzulu Hospital, Durban, South Africa; 2 Division of Pulmonology, Department of Medicine, Faculty of Health Sciences, University of Cape Town and Groote Schuur Hospital, Centre for the AIDS Programme of Research in South Africa, Cape Town, South Africa

**Keywords:** drug-resistant tuberculosis, adjuvant lung resection, thoracic surgery, multidisciplinary teams

## Abstract

**Background:**

Adjuvant lung resection in patients with drug-resistant tuberculosis (DR-TB) not only is cheaper than a 2-month course of drug therapy for multidrug-resistant tuberculosis (MDR-TB) but also, more importantly, has a higher cure rate than medical therapy alone. The cure rate for some MDR-TB patients treated with adjuvant lung resection is about 90%. With the more severe forms of DR-TB, surgical cure rates in selected patients remain high, whereas cure rates decrease when only medical therapy is used. In addition, adjuvant lung resection for DR-TB in selected patients with HIV co-infection does not appear to have a higher complication rate.

**Objectives:**

To determine whether specialist pulmonologists in South Africa utilise thoracic surgical intervention for DR-TB appropriately.

**Methods:**

A cross-sectional survey was conducted among pulmonologists of the South African Thoracic Society. The study tool was a predesigned, anonymous questionnaire that included 17 closed-ended questions about the role of cardiothoracic surgery in the management of DR-TB.

**Results:**

A 50% response rate was achieved. The majority of respondents did not know the indications for adjuvant lung surgery in the setting of DR-TB, but would have altered their referral behaviour had they been aware of these indications.

**Conclusion:**

Participating pulmonologists’ uncertainty regarding optimal use of adjuvant lung resection for DR-TB suggests the need for local guidelines and education initiatives relevant to the management of these patients. These strategies should include the participation of both the pulmonologist and the cardiothoracic surgeon.

## Background

Globally, tuberculosis (TB) is the leading cause of death due to a curable infectious disease and was ranked as the leading cause of death in South Africa (SA) already in 2015.^[1,2]^ As a disease of antiquity, it has remained an insurmountable global health challenge and the emergence of drug-resistant tuberculosis (DR-TB) threatens to discount the considerable gains we have made towards disease control and eradication. TB will cost the world’s poorest countries up to USD3 trillion over the next 10 years and the World Bank estimates the loss of productivity attributable to TB at 4% - 7% of some countries’ GDP, as 75% of TB cases occur in patients between 15 and 54 years of age.^[3]^

In SA, the directly observed treatment short-course (DOTS) strategy achieves a 67% cure rate while disease recurrence is alarmingly high.^[4,5]^ The combination of disease recurrence, unrestrained disease transmission, limited access to diagnostic services and the absence of highly effective treatment options has fuelled the DR-TB epidemic.^[6,7]^

In SA, medical therapy alone achieves a 6-month culture conversion rate of less than 20% in HIV-infected patients with extensively drug-resistant tuberculosis (XDR-TB).^[8]^ In 2013, the World Health Organization stated that about 20% of the 94 000 multidrug-resistant tuberculosis (MDR-TB) cases diagnosed in 2012 went untreated.^[9,10]^

Newer drugs such as delamanid, pretomanid and bedaquiline have recently been included in the medical armamentarium, but access to the drugs remains restricted. There is promising evidence that a new standardised regimen for the treatment of MDR-TB may offer the benefits of a shorter treatment duration with improved treatment success.^[11]^ Medical therapy alone for the treatment of MDR-TB has been shown to achieve a cure rate of approximately 54%, while newer medical treatment strategies demonstrate the potential to achieve higher cure rates.^[11,12]^ However, HIV-positive patients were largely excluded from trials of newer drugs and bedaquiline, which was associated with a higher mortality, has been recommended for use only when there is a lack of four effective drugs or when MDRTB is accompanied by fluoroquinolone resistance.^[13,14]^ Despite the introduction of newer and repurposed drugs, only 80% of patients with MDR-TB and about 40% of those with XDR-TB may be cured with appropriate medical therapy alone.^[11]^ Adjuvant lung resection in selected patients has the potential to improve these treatment outcomes further.^[8,15-17]^ The cure rate for selected MDR-TB patients is about 90% with adjuvant lung resection.^[18]^

In a study by Gandhi *et al*.,^[5]^ 52 of 53 XDR-TB patients (98%) died, with a mean survival of 16 days from the time of specimen collection to death. Of the 44 patients with XDR-TB who consented to testing for HIV, all were found to be co-infected. Although prompt initiation of antiretroviral therapy (ART) in these patients has been demonstrated to improve their outcome, cure rates remain suboptimal.^[18]^ Once again, adjuvant lung resection has an important role in improving the gains of effective medical therapy in this context.^[16]^

The emergence of adjuvant lung resection in the management of DRTB will require multidisciplinary teams to identify suitable candidates and to provide pre-, intra- and postoperative care. Such teams might consist of pulmonologists, cardiothoracic surgeons, infectious disease specialists, microbiologists and allied health professionals. As physicians who are entrusted with the medical care of complicated DR-TB patients, pulmonologists will likely be required to initiate a referral to such a multidisciplinary platform. In this survey, we explore the knowledge and practices of SA pulmonologists predominantly with regard to the role of adjuvant lung resection for the management of DR-TB.

## Methods

A cross-sectional survey of all pulmonologists of the South African Thoracic Society was conducted. The South African Thoracic Society is the largest representative professional society of pulmonologists in southern Africa. A list of all registered pulmonologists was made available by the Society and all pulmonologists received an electronic invitation to participate in the survey. The study tool was a predesigned, anonymous questionnaire that included 17 closed-ended questions regarding the role of cardiothoracic surgery in the management of DR-TB. As the study was purely descriptive and no comparisons were made, no further statistical evaluation was performed.

## Results

A response rate of 50% was achieved, with 25 pulmonologists responding to the survey. The distribution of responses is reported as percentages (Table 1). Approximately three-quarters (76%) of respondents saw fewer than 10 DR-TB patients per year, while the remaining 24% saw between 10 and 50 DR-TB patients per year. In addition, 80% of respondents reported having no access to novel drug therapy for their patients. Almost two-thirds (64%) of respondents knew the indications for lung resection associated with DR-TB, although a large percentage (60%) underestimated the potential cure rates for DR-TB with adjuvant lung resection. More than a third (36%) of the respondents did not recognise the inferior outcomes of medical therapy alone in optimally treated HIV-infected patients compared with HIV-negative patients, and one-fifth (20%) of respondents considered adjuvant lung resection to be associated with poorer outcomes in optimally treated HIV-infected patients than in HIV-negative patients. The majority of respondents (88%) indicated that, having been informed of the role of thoracic surgery in the management of DR-TB, they would consider referring their DR-TB patients for surgical assessment in future, despite 92% never having done so in the past; in this survey, only 8% of respondents referred every DR-TB patient for a surgical evaluation. Only 32% of respondents knew what the appropriate timing was for referring patients for surgical assessment after initiating effective drug therapy, while the remainder indicated that such a referral could be made only after at least 6 months. Flexible bronchoscopy, mediastinoscopy, scalene node biopsy and thoracoscopy were the most commonly used invasive thoracic diagnostic modalities used for the diagnosis of DR-TB.

**Table 1:** Survey questions and associated responses

**Question**	**Response, *n*(%)**
**Do you know the indications for lung resection for DR-TB?**	
Yes	16 (64)
No	9 (36)
**What is the cure rate for MDR-TB with adjuvant lung resection?**	
30%	3 (12)
50%	12 (48)
90%	10 (40)
**Do HIV-positive patients on highly active antiretroviral therapy (HAART), with an undetectable viral load and normal nutrition, have lower cure rates for DR-TB with medical therapy alone?**	
Yes	9 (36)
No	16 (64)
**Do HIV-positive patients on HAART, with an undetectable viral load and normal nutrition, have lower cure rates for DR-TB with adjuvant lung resection?**	
Yes	5 (20)
No	20 (80)
**Listed below are the indications for adjuvant lung resection for DR-TB:**	
High risk of treatment failure/relapse	
2 or more relapses/1 or more relapses while on treatment	
Persistent sputum positivity after 3 - 6 months of therapy and within 2 months of surgery	
Persistent cavitatory disease	
Intolerance to medical therapy	
Localised disease (absolute criterion)	
Bronchiectasis and complications	
Haemoptysis	
**Would knowing these indications influence your referral for surgery in the future?**	
Yes	22 (88)
No	3 (12)
**Did you refer every patient with DR-TB for surgical assessment?**	
Yes	2 (8)
No	23 (92)
**Would your referral for surgery be negatively influenced if, after appropriate drug therapy, the patient remains culture positive?**	
Yes	23 (92)
No	2 (8)
**What is the recommended duration of drug therapy for DR-TB prior to lung resection? (months)**	
<3	1 (4)
3 - 6	7 (28)
6 - 12	4 (16)
12 - 18	7 (28)
18 - 24	6 (24)
>24	0 (0)
**What is the bacillary load content of pulmonary TB cavities?**	
0	0
<100	0
10^2^ - 10^4^	12 (48)
10^7^ - 10^9^	13 (52)
**What is the bacillary load content of pulmonary TB nodules?**	
0	0
<100	12 (48)
10^2^ - 10^4^	10 (40)
10^7^ - 10^9^	3 (12)
Indicate the diagnostic techniques listed below that you have utilised for the diagnosis of TB:*	
Flexible bronchoscopy	24 (96)
Rigid bronchoscopy	7 (28)
Mediastinoscopy	12 (48)
Anterior scalene lymph node biopsy	8 (32)
Anterior mediastinotomy	6 (24)
VATS lung biopsy	7 (28)
Open lung biopsy	2 (8)
Thoracoscopy	9 (36)
Open pleural biopsy	8 (32)
**Is there a significant difference in surgical complications between lung resection for DR-TB and that for DS-TB or sequelar TB?**	
Yes	10 (40)
No	15 (60)
**Would you agree that adjuvant surgery may potentially allow the effective implementation of decentralisation programmes and decreased duration of DR-TB therapy?**	
Yes	21 (84)
No	4 (16)
**How many patients do you see per annum with DR-TB?**	
<10	19 (76)
10 - 50	6 (24)
>50	0
**In the last 5 years of practice, how many patients have you referred for an opinion for lung resection for DR-TB?**	
<10	23 (92)
10 - 50	2 (8)
>50	0
**Does your laboratory routinely analyse for capreomycin resistance?**	
Yes	1 (4)
No	24 (96)
**Are novel drugs such as bedaquiline or linezolid easily available to your patients?**	
Yes	5 (20)
No	20 (80)

DR-TB = drug-resistant tuberculosis

MDR-TB = multidrug-resistant tuberculosis

VATS = video-assisted thoracoscopic surgery

DS-TB = drug-sensitive tuberculosis

TB = tuberculosis

*It is essential to remember that bronchial washings have a far lower yield than bronchoalveolar lavage, which is usually undertaken utilising general anaesthesia.

## Discussion

Despite the worsening DR-TB epidemic, there appears to be no effective multidisciplinary management approach in SA. This survey highlights the ineffective utilisation of curative adjuvant lung resection in selected DR-TB patients.

Only 64% of the respondents were familiar with the indications for adjuvant lung resection associated with DR-TB. Moreover, about half of the respondents were not aware of the cure rate associated with adjuvant lung resection for MDR-TB. It stands to reason that because respondents were unaware of the benefits of adjuvant lung resection, 92% did not refer their DR-TB patients for a surgical opinion. Unfortunately, no studies have focused on a multidisciplinary approach in treating patients with DR-TB.

There is an alarmingly high mortality with medical therapy alone in patients with DR-TB/HIV co-infection and especially in those with XDR-TB.^[5]^ Although the majority of respondents agreed that adjuvant lung resection is not associated with lower cure rates for DR-TB in HIV-negative patients than in HIV-positive patients who receive ART and who have an undetectable viral load and normal nutrition, this treatment option again appears to have been underutilised. A retrospective study reviewing adjuvant lung resection in patients co-infected with DR-TB/HIV showed cure rates of 75% and 62%, and conversion rates of 22% and 38% in the MDR-TB/HIV-positive group and the XDR-TB/HIV-positive group, respectively.^[15]^ Furthermore, it has been shown that lung resection for massive haemoptysis, whether undertaken electively or emergently and irrespective of a patient’s HIV status, does not have a significantly higher complication rate. The CD4 cell count or viral load was considered only in elective cases.^[15]^ In patients who were HIV positive and not on ART, the CD4 cell count had to be >200 cells/µL prior to lung resection; in patients on ART, the viral load had to be undetectable prior to lung resection.^[15]^ Preoperative risk factors such as previous opportunistic lung infections, decreased effort tolerance, a low CD4 cell count without ART, persistently elevated viral load despite ART and nutritional status (in particular, an albumin level <30 g/dL) may preclude major thoracic surgery. Appropriately selected patients with DR-TB and HIV co-infection may undergo major thoracic surgery with the anticipation of good outcomes.^[19]^

In this survey, 92% of respondents stated that referral for surgery would be negatively influenced if, after appropriate drug therapy, the patient remains culture positive. It is recommended that patients in whom the isolate is relatively resistant to all antibiotics undergo lung resection within the first 2 months, if feasible. Others should have medical therapy for at least 3 months. After 3 months of medical therapy, the sputum cultures will either be TB-culture negative or have a minimal mycobacterial count.^[20]^ It is essential to undertake surgery before the count rises and it is for these reasons that lung resection is undertaken at this point. An unnecessary delay in surgery may result in the disease spreading beyond the reach of curative surgical resection.

The indications for surgery for DR-TB include: high risk of treatment failure/relapse; 2 or more relapses/1 or more relapses while on treatment; persistent sputum positivity after 3 - 6 months of therapy within 2 months of surgery; persistent cavitatory disease; intolerance to medical therapy; localised disease equivalent to one lung (absolute criterion); haemoptysis; and bronchiectasis.^[20,21]^

Medical therapy should continue for at least 2 years following surgery.^[21]^ XDR-TB, which occurs in a large proportion of MDR-TB patients, was an independent poor prognostic factor predictor even in HIV-negative patients.^[22,23]^

It has been established that the bacillary load content of pulmonary TB cavities is 10^7^ - 10^9^ and that of pulmonary TB nodules is 10^2^ - 10^4^ bacilli, yet only 52% and 40% of respondents, respectively, correctly acknowledged these facts.^[20]^ As such, patients with lung cavities and nodules are at a high risk of recurrence, even if sputum-negative. Moreover, drug penetration of the cavities and nodules is suboptimal.

Despite excellent results with adjuvant lung resection, albeit in selected patients, medical therapy still remains a virtually exclusive form of management among most of the physicians who participated in this survey. This appears to be the exclusive result of a lack of knowledge regarding the benefits of and indications for adjuvant lung resection. We suggest that a national multidisciplinary team be established to formalise treatment guidelines for adjuvant lung resection in DR-TB patients, irrespective of HIV status. This should include patient selection and timing of surgery based on current practices in the various cardiothoracic units in SA. Surgical outcomes in the various units making proposals should be taken into account.

It is the authors’ opinion that such guidelines should be formally endorsed by the Department of Health, once established. All units treating DR-TB patients should comprise a mandatory multidisciplinary team. Every opportunity should be used to ensure that these guidelines are accessible to the relevant healthcare practitioners, for example through TB awareness road shows, media campaigns, journal publications and local congresses.

A national DR-TB register to collect relevant surgical data is also recommended. It should include standardised radiological reports, reasons for proposing or declining adjuvant lung resection and a record of surgical outcomes. Such a registry would allow participation from both the public and the private sectors.

### Study limitations

The small number of respondents is an obvious limitation and the results of this study may not be generalisable to other settings. Furthermore, the small sample size precluded further statistical evaluation and the identification of potentially useful predictors of practitioner perceptions or behaviour.

## Conclusion

DR-TB is, and will continue to be, a formidable adversary, which requires role players to deal collectively, effectively and timeously with patients presenting with this disease. The uncertainty regarding optimal utilisation of adjuvant lung resection for DRTB patients among pulmonologists suggests the need for educational strategies relevant to the optimal management of these patients. These strategies should include the participation of both the pulmonologist and the cardiothoracic surgeon. This survey further highlights the lack of a multidisciplinary approach to effectively implement appropriate management of DR-TB.
